# Early Post-STEMI Cardiac Rehabilitation in the CSC-Infarct Program: Real-World Safety and Effectiveness of Individualized Training Protocols

**DOI:** 10.3390/jcm15020746

**Published:** 2026-01-16

**Authors:** Agnieszka Grochulska, Sebastian Glowinski, Aleksandra Bryndal

**Affiliations:** 1 Department of Physiotherapy, Institute of Health Sciences, Pomeranian University in Słupsk, Westerplatte 64, 76-200 Słupsk, Poland; 2 Institute of Physical Culture and Health, State University of Applied Sciences in Koszalin, Leśna 1, 75-582 Koszalin, Poland

**Keywords:** cardiac rehabilitation, STEMI, remodeling, Coordinated Specialist Care, interval training, continuous training, early rehabilitation, cardiorespiratory fitness

## Abstract

**Background/Objectives**: Cardiac remodeling post-myocardial infarction is a critical process determining patient prognosis. Poland’s Coordinated Specialist Care program enables early cardiac rehabilitation (CSC-Infarct) during peak remodeling period. This study evaluated the safety and effectiveness of very early cardiac rehabilitation initiated during peak remodeling (mean 16.8 ± 3.4 days post- ST-elevation myocardial infarction [STEMI]) within the CSC-Infarct program. We examined outcomes following two training modalities—interval and continuous—applied according to clinical guidelines based on baseline exercise capacity. **Methods**: We enrolled 288 patients (135 women, 153 men, age 59.7 ± 9.8 years) after first STEMI into a 24-day rehabilitation program (5 sessions/week) within CSC-Infarct. Patients received either interval training (n = 127) or continuous training (n = 161) according to National Health Fund protocols. Hemodynamic, metabolic (metabolic equivalents [MET], maximal oxygen uptake [VO2max]), and functional parameters (6-minute walk test [6MWT]) were assessed pre- and post-rehabilitation. **Results**: Both groups showed significant improvement in most parameters. The continuous training group achieved higher final MET values (8.9 ± 2.5 vs. 6.5 ± 1.9; *p* < 0.001), VO_2_max (31.0 ± 8.8 vs. 22.9 ± 6.5 mL/kg/min; *p* < 0.001), and 6MWT distance (530.9 ± 108.9 vs. 455.6 ± 104.3 m; *p* < 0.001). Significant improvement in heart rate recovery (HRR), indicating autonomic balance, was observed only in the continuous training group (*p* = 0.026), not in the interval group (*p* = 0.290). **Conclusions**: Early rehabilitation within CSC-Infarct (mean 16.8 days post-infarction) during intensive remodeling is safe and effective. Both training modalities produced clinically significant improvements when appropriately matched to patient baseline capacity. Continuous training showed additional benefit in autonomic balance (HRR improvement), while interval training achieved substantial relative gains (+11.8% in 6MWT) in lower-capacity patients. The CSC-Infarct program provides optimal timing for rehabilitation implementation during the critical cardiac remodeling period.

## 1. Introduction

Cardiovascular diseases remain the leading cause of death worldwide, accounting for 17.9 million deaths globally in 2019 [1]. In Poland in 2018, over 26,700 cases of ST-elevation myocardial infarction (STEMI) were recorded, with 30-day mortality of approximately 9.0% [2]. Despite advances in invasive and pharmacological treatment, STEMI remains a serious public health problem due to the risk of heart failure development and premature death [3,4].

Following myocardial infarction, a complex process of left ventricular remodeling occurs, progressing through three overlapping phases: inflammatory (0–7 days), proliferative (7–21 days), and scar maturation (3 weeks–several months) [5,6]. During the inflammatory phase, neutrophil and macrophage infiltration occurs, along with initiation of extracellular matrix (ECM) degradation [7,8]. Matrix metalloproteinases (MMPs), particularly MMP-2 and MMP-9, play a key role in this process, with peak activity occurring within the first 7 days after infarction [9,10,11,12,13]. Imbalance in MMP activity may result in left ventricular dilatation and progression to heart failure [14,15,16,17,18,19,20,21].

Cardiac rehabilitation is an integral element of cardiovascular disease therapy with the highest class of recommendation (Class I, Level A) in ESC guidelines for post-myocardial infarction patients [22,23]. Early initiation of rehabilitation, still during hospitalization or within 2–3 weeks after infarction, is particularly important in the context of remodeling processes [24]. Studies show that early rehabilitation intervention initiated within 14–21 days after infarction—that is, during the phase of most intense remodeling—can favorably influence this process through modulation of MMP expression and limitation of adverse left ventricular dilatation [16,17,25,26].

Physical training does not cause adverse changes in remodeling and may improve left ventricular ejection fraction (LVEF) and reduce ventricular dimensions [27,28,29]. The mechanisms of this protection likely include modulation of inflammatory response, influence on MMP/TIMP balance, and improvement in cardiovascular autonomic function [30,31].

In Poland, access to cardiac rehabilitation remains concerningly low, with average waiting time for rehabilitation reimbursed by the National Health Fund (NFZ) reaching up to 12 months [32]. In 2019, only 5% of acute coronary syndrome (ACS) cases underwent rehabilitation within 14 days of admission, 25% within 60 days, and 28% within 90 days, representing an increase compared to 2014 but still an unsatisfactory result [33]. Long waiting times adversely affect the effectiveness of the therapeutic process, as the later the appropriate therapy is implemented, the greater the risk of complications and the more difficult the implementation of secondary prevention.

The response to this problem was the launch in 2017 of the Coordinated Specialist Care Program—Myocardial Infarction (CSC-Infarct), financed by NFZ [34,35]. This program provides post-STEMI patients with comprehensive care, including early cardiac rehabilitation initiated on average within 2–3 weeks after infarction [31]. Within CSC-Infarct, patients have a guaranteed 24-day outpatient rehabilitation program (5 sessions per week) according to standards defined by NFZ [32]. Rehabilitation includes physical training (interval or continuous) tailored to individual patient capacity, health education, and monitoring of cardiovascular risk factors [34,35,36,37].

Early initiation of rehabilitation within CSC-Infarct falls during the period of most intense cardiac remodeling, which may be crucial for long-term prognosis. Previous studies indicate the safety of early rehabilitation; however, the optimal training modality (interval versus continuous) during this critical period has not yet been fully determined [36,37,38,39,40,41,42].

Interval training is characterized by alternating periods of higher-intensity exercise and recovery, which may be beneficial for patients with lower exercise tolerance [39]. Conversely, continuous training provides steady, moderate exercise throughout the training session, which may lead to different physiological adaptations of the cardiovascular system [43,44].

The primary aim of this study was to evaluate the safety and effectiveness of very early cardiac rehabilitation initiated within the CSC-Infarct program (mean 16.8 ± 3.4 days post-STEMI) during the critical cardiac remodeling period. Secondarily, we describe outcomes following two training modalities—interval and continuous—as implemented according to clinical practice guidelines based on baseline exercise capacity. This pragmatic, observational design reflects real-world clinical decision-making where training intensity is individualized to patient characteristics and safety considerations.

Specifically, we examined cardiorespiratory fitness (VO_2_max, MET), functional capacity (6 min walk test), hemodynamic parameters (heart rate, blood pressure), and autonomic balance indices (heart rate recovery) in patients rehabilitated during the inflammatory-proliferative transition phase of remodeling.

The following hypotheses were formulated: (1) very early rehabilitation initiated during peak cardiac remodeling (16–17 days after infarction) will be safe, with no serious adverse events; (2) early rehabilitation will produce clinically meaningful improvements in cardiorespiratory and functional capacity regardless of training modality when protocols are appropriately matched to baseline exercise tolerance; (3) both training modalities will demonstrate favorable effects on cardiovascular parameters within their respective target populations.

## 2. Materials and Methods

### 2.1. Study Design and Participants

A retrospective observational study was conducted from April 2019 to June 2025 at the Cardiac Rehabilitation Center of the Janusz Korczak Provincial Specialist Hospital in Słupsk. Participants were patients enrolled in the Coordinated Specialist Care Program—Myocardial Infarction (CSC-Infarct) after ST-elevation myocardial infarction (STEMI) treated with primary percutaneous coronary intervention (PCI). Sample size was determined by consecutive enrollment of all eligible patients during the study period. The study included a total of 288 patients (135 women, 153 men).

All patients initiated cardiac rehabilitation within (CSC-Infarct) on average 16.8 ± 3.4 days after infarction, corresponding to the period of intense left ventricular remodeling. This timeframe encompasses the inflammatory phase and the beginning of the proliferative phase, when MMP activity is particularly high and rehabilitation intervention may have the greatest impact on the healing process of damaged cardiac muscle [5,6].

### 2.2. Inclusion and Exclusion Criteria

Inclusion criteria: (1) first STEMI in medical history; (2) successful primary PCI (TIMI flow 2 or 3); (3) stable clinical condition; (4) left ventricular ejection fraction (LVEF ≥ 35%); (5) enrollment in the (CSC-Infarct) program; (6) informed written consent to participate in the study. Exclusion criteria: (1) unstable coronary artery disease; (2) ventricular arrhythmias; (3) severe heart failure (NYHA IV—New York Heart Association); (4) uncompensated organ failure; (5) musculoskeletal diseases preventing training; (6) lack of consent to participate in the study.

### 2.3. Rehabilitation Protocol

The cardiac rehabilitation program was implemented according to the guidelines of the Section of Cardiac Rehabilitation and Exercise Physiology of the Polish Cardiac Society and NFZ standards [43,44], which are consistent with current international recommendations from the ESC (European Society of Cardiology) (2024) and AHA (American Heart Association) (2024) [22,23]. All patients received guideline-directed medical therapy [22,23,26] including beta-blockers. Pharmacological treatment remained stable throughout rehabilitation, consistent with stable resting heart rate. The program included 24 intensive training sessions conducted over 5 weeks (5 sessions per week) [34,35]. Each session lasted 60–90 min and consisted of: (1) warm-up (10 min); (2) endurance training on cycle ergometer (30 min); (3) general development exercises—including respiratory muscle training, stretching and flexibility exercises (20 min); (4) resistance training with light loads (15 min); (5) relaxation (5–10 min) [3,4,43,44,45].

Patients were assigned by a cardiologist and physiotherapist to the appropriate training protocol based on baseline exercise tolerance determined in exercise testing according to the Bruce protocol, following the center’s standard clinical protocol and based on analysis of atherosclerosis risk factors and cardiac events during physical training. Patients with exercise tolerance (MET ≥ 6), good physical exercise tolerance, and low risk of cardiac events during physical training were assigned to continuous training (n = 161). Patients with exercise tolerance (MET < 6), moderate to low physical exercise tolerance, and moderate to high risk of cardiac events during physical training were assigned to interval training (n = 127).

#### Clinical Rationale for Training Assignment

Training modality assignment reflected standard clinical practice in Polish cardiac rehabilitation centers, consistent with Polish Cardiac Society guidelines and international recommendations (ESC 2024, AHA 2024) [22,23,43,44]. This individualized approach ensures patient safety and optimal therapeutic benefit by matching training intensity to individual exercise capacity and clinical risk profile.

Patients assigned to interval training typically present with lower baseline capacity (MET < 6) and may have additional factors (advanced age, multiple comorbidities, moderate-high clinical risk) warranting a more conservative rehabilitation approach with alternating periods of exercise and recovery. This protocol allows safe participation and gradual cardiovascular adaptation in patients who might not tolerate sustained higher-intensity exercise.

Conversely, continuous training is clinically appropriate for patients demonstrating higher exercise tolerance (MET ≥ 6) and lower clinical risk, who can safely benefit from sustained moderate-intensity exercise that maximizes aerobic adaptation and cardiovascular conditioning. This allocation strategy is not a source of bias but rather represents appropriate clinical care tailored to individual patient needs, ensuring both safety and therapeutic efficacy across the full spectrum of post-STEMI patients requiring rehabilitation.

### 2.4. Clinical Parameter Assessment

All baseline measurements were performed 2–3 days before starting the rehabilitation program. Post-rehabilitation assessments were conducted 2–3 days after completing the 24-day program. The assessment included: (1) Exercise testing on a treadmill according to the Bruce protocol with continuous ECG recording (only before starting the rehabilitation program), blood pressure measurement and assessment of hemodynamic parameters (HR rest, HR max, HRR, blood pressure); (2) Estimation of physical capacity through MET measurement and calculation of VO_2_max according to the formula: VO_2_max = MET × 3.5 (mL/kg/min) [8]; (3) 6 min walk test (6MWT); (4) Assessment of subjective perception of exertion using the Borg scale (6–20 points); (5) Anthropometric measurements (height, body weight, body mass index [BMI], waist-to-hip ratio [WHR]); (6) Echocardiography with LVEF assessment. Clinical parameters were assessed based on the protocol described in detail in our previous work [8,46,47].

### 2.5. Statistical Analysis

Statistical analysis was performed using the Statistica 13.3 package (TIBCO Software Inc., Palo Alto, CA, USA). Continuous data were presented as mean and standard deviation, median, and 95% confidence interval (95% CI) calculated for the mean. Selection of statistical tests depended on the nature of the data: normality of distribution was assessed before analysis (Shapiro–Wilk test), and its result determined the application of parametric or nonparametric procedures. For normally distributed data, Student’s *t*-test for independent or dependent samples was used; when variance inequality occurred, Welch’s correction was applied. For data not meeting the normality criterion, the Mann–Whitney U test (comparisons between groups) and Wilcoxon test (pre-post comparisons) were used. Categorical variables were analyzed using chi-square test with Yates’ correction. The level of statistical significance was set at *p* < 0.05. Because training modality was assigned based on baseline exercise capacity and clinical risk, between-group comparisons were interpreted descriptively. No ANCOVA or multivariable adjustment was applied, as baseline MET constituted the main allocation criterion and was strongly collinear with outcome variables, violating key assumptions for covariate adjustment. Analyses included comparison of hemodynamic, metabolic, and functional parameters between continuous and interval training groups, as well as assessment of rehabilitation effects within the CSC-Infarct program. Results were presented in tabular and graphical form, including heart rate, blood pressure, MET, VO_2_max, 6 min walk test, and double product, enabling comprehensive assessment of the safety and effectiveness of rehabilitation interventions.

Important Note on Study Interpretation: While we present between-group statistical comparisons, these should be interpreted as descriptive characterizations of outcomes in different patient populations rather than as causal comparisons of training efficacy. The observational nature of our study, with indication-based rather than random allocation, means that observed differences reflect both the training interventions AND the baseline characteristics of patients appropriately assigned to each protocol. Our primary conclusions concern the safety and effectiveness of very early rehabilitation timing (16–17 days post-STEMI) regardless of protocol, rather than superiority of one training method over another. Direct causal comparison of training modalities would require a randomized controlled trial with matched baseline characteristics, which was not the objective of this pragmatic, real-world evaluation.

### 2.6. Ethical Considerations

The study was conducted in accordance with the Declaration of Helsinki and approved by the Bioethics Committee at the Regional Medical Chamber in Gdansk (No. KB-17/16). Each patient provided written informed consent to participate in the study after receiving detailed information about its purpose and course. All patients had the right to withdraw consent at any time without stating a reason. Patient data were anonymized and stored in accordance with GDPR requirements.

## 3. Results

### 3.1. Study Group Characteristics

**Figure 1:**
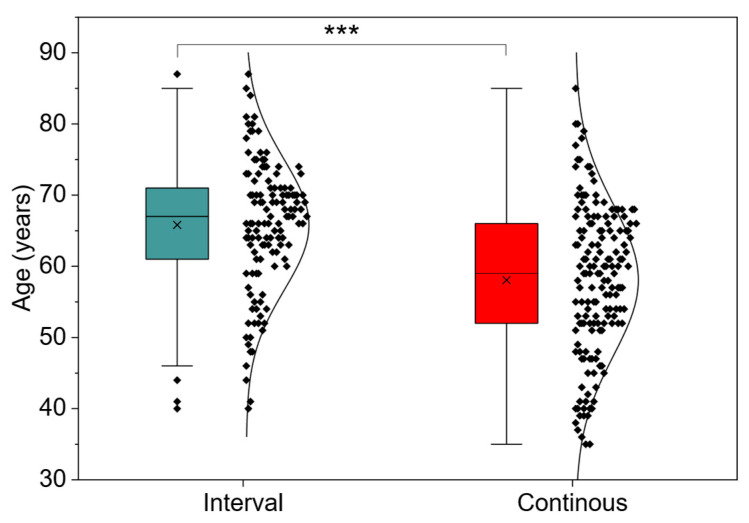
Patient age in years by interval and continuous training (N = 288), ***—*p* < 0.001.

Table 1 presents the demographic and clinical characteristics of 288 patients enrolled in early cardiac rehabilitation within the CSC—Infarct program, divided into interval training group (n = 127) and continuous training group (n = 161). Both groups were comparable in terms of sex, anthropometric parameters (BMI, WHR), and left ventricular ejection fraction. A significant difference was found only in age—patients assigned to interval training were older (65.8 ± 9.2 years) than continuous training participants (58.1 ± 10.6 years; *p* = 0.001) (Figure 1). Time from infarction to rehabilitation initiation was short and similar in both groups (average 16.8 ± 3.4 days), which emphasizes the homogeneity of intervention timing. Despite the age difference, the groups remained balanced in terms of key clinical parameters, enabling reliable comparison of the effects of both training protocols.

Analysis of cardiovascular risk factors (Table 2) confirmed their high prevalence in the studied population, which is consistent with observations regarding Polish post-infarction patients [42]. Overweight or obesity was found in 64.2% of patients, hypertriglyceridemia in 65.3%, smoking in 39.2%, hypertension in 76.0%, and diabetes in 37.9%. The only significant difference between groups was the more frequent presence of hypertension in the interval training group (86.6% vs. 67.7%; *p* < 0.001) [48,49].

### 3.2. Rehabilitation Outcomes by Training Protocol

Both training protocols, when applied to their clinically appropriate populations, resulted in significant improvements across multiple parameters. The following analysis characterizes outcomes in patients assigned to each protocol based on baseline exercise capacity and clinical risk profile. Between-group differences in final values reflect both differential training responses and baseline patient characteristics that determined protocol assignment.

After completing the 24-session program, rehabilitation effects were assessed in both training groups (Table 3). Both groups achieved significant improvement in most parameters; however, final values differed, reflecting both different baseline fitness levels, which formed the basis for protocol assignment and potential differences in exercise response.

Regarding hemodynamic parameters, patients in the continuous training group achieved higher maximum heart rate (HR max) compared to the interval group (119.4 ± 17.0 vs. 114.5 ± 16.4 beats/min; *p* = 0.029). Resting heart rate did not differ significantly between groups either before or after rehabilitation completion. Analysis of heart rate recovery (HRR) proved particularly significant. Although final HR at 1 min post-exercise was comparable (86.7 ± 18.5 vs. 87.9 ± 15.1; *p* = 0.538), HRR improvement was observed only in the continuous group (*p* = 0.026), while in the interval group the change was not significant (*p* = 0.290), which may reflect more favorable autonomic adaptation to exercise.

Regarding metabolic parameters and physical capacity indices, the continuous group achieved higher final values for MET (8.9 ± 2.5 vs. 6.5 ± 1.9; *p* < 0.001), VO_2_max (31.0 ± 8.8 vs. 22.9 ± 6.5 mL/kg/min; *p* < 0.001), and percent of predicted exercise capacity (107.8 ± 27.7% vs. 94.6 ± 25.3%; *p* < 0.001). These differences should be interpreted in the context of different baseline values, consistent with protocol assignment criteria (MET: 7.0 ± 1.5 vs. 4.9 ± 1.6; *p* < 0.001).

In the 6MWT, the continuous training group achieved a significantly better final result (530.9 ± 108.9 vs. 455.6 ± 104.3 m; *p* < 0.001). The 75 m difference exceeds the minimal clinically important difference (30–50 m) for patients after acute coronary syndrome, confirming the clinical significance of the improvement achieved.

Double product (DPr), an indirect indicator of myocardial oxygen demand, was comparable between groups after rehabilitation completion (18484 ± 3880 vs. 17916 ± 4036; *p* = 0.3368). Subjective perception of exertion assessed using the Borg scale significantly decreased in both groups, with final values lower in the continuous group (12.8 ± 0.9 vs. 13.5 ± 0.9; *p* < 0.001). Blood pressure parameters and arterial blood saturation remained stable and did not differ significantly between groups at any measurement point.

Analysis of within-group changes before and after rehabilitation (Table 4) demonstrated significant improvement in most hemodynamic, metabolic, and functional parameters in both training groups. In the entire study population, a significant increase in maximum heart rate (HR max; *p* < 0.001) was observed, which was also confirmed in subgroup analyses—both in the interval and continuous groups (*p* < 0.001 for both). Resting heart rate remained stable, which is consistent with the effect of beta-blocker therapy.

An important observation concerned differences in heart rate recovery (HRR). In the entire group, no significant change in HR at 1 min post-exercise was noted (*p* = 0.252). In subgroup analysis, HRR improvement was found only in patients performing continuous training (*p* = 0.026), while in the interval group the change was not significant (*p* = 0.290), which may reflect more effective autonomic adaptation in this form of training.

Metabolic parameters showed unequivocally favorable changes. MET value increased significantly in the entire group (*p* < 0.001) and in both subgroups (*p* < 0.001 for each). Similarly, percent of predicted exercise capacity for age and VO_2_max improved significantly both in the entire population and in subgroups (*p* < 0.001 in all cases). Double product (DPr), reflecting myocardial oxygen demand, also increased in both training protocols (*p* < 0.001).

In the 6 min walk test (6MWT), both groups achieved gains exceeding the minimal clinically important difference (30–50 m). The improvement was 48.0 m (+11.8%) in the interval group and 41.5 m (+8.5%) in the continuous group. Although final values were higher in the continuous group, within-group analysis confirms the clinical effectiveness of both protocols.

Subjective perception of exertion on the Borg scale decreased significantly in the entire population (*p* < 0.001) and in each subgroup. Blood pressure parameters remained stable, except for a significant decrease in diastolic blood pressure during exercise, observed both in the entire group (*p* < 0.001) and in both subgroups. Additionally, arterial blood saturation increased significantly in the entire population and in each analyzed subgroup (*p* < 0.001), confirming the safety and effectiveness of the rehabilitation conducted.

**Figure 2:**
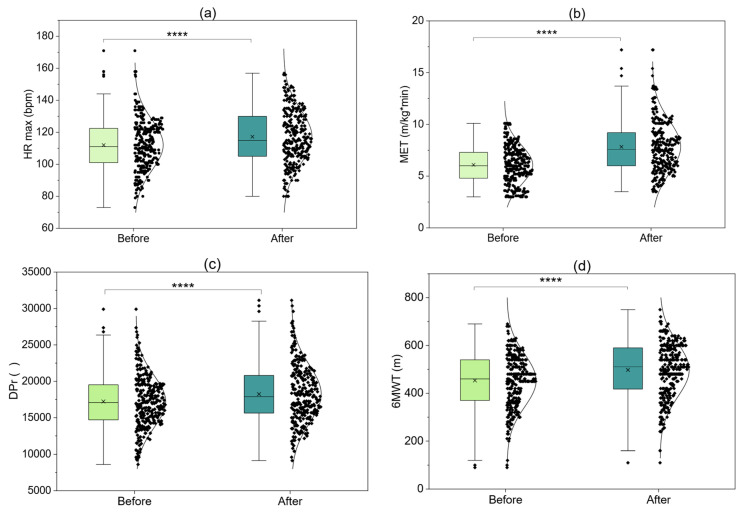
Baseline and final assessments of all patients combined (without division by therapy type) before and after therapy (**a**) HR max; (**b**) MET; (**c**) DPr; (**d**) 6 MWT; ****—*p* < 0.0001.

Figure 2 presents four panels illustrating the comparison of baseline and final values for the entire patient group (N = 288), encompassing important hemodynamic, metabolic, and functional parameters. Figure 2a indicates that after rehabilitation, a significant increase in maximum heart rate achieved during exercise (HR max) was observed. This signifies improved exercise tolerance and greater ability to achieve higher physical workloads. Figure 2b illustrates the metabolic equivalent (MET) value, which increased significantly after program completion. This reflects increased physical capacity and improvement in the body’s aerobic capabilities. Figure 2c presents DPr (double product), an indirect indicator of myocardial oxygen demand. This parameter also showed a significant increase. This increase is interpreted as an effect of improved cardiac capacity to work under greater load with reduced hemodynamic risk. Figure 2d presents the distance covered in the 6 min walk test (6MWT). It increased significantly, demonstrating clear improvement in functional capacity and exercise tolerance in daily life. All four panels show highly statistically significant differences (*p* < 0.001), confirming the effectiveness of early cardiac rehabilitation.

**Figure 3:**
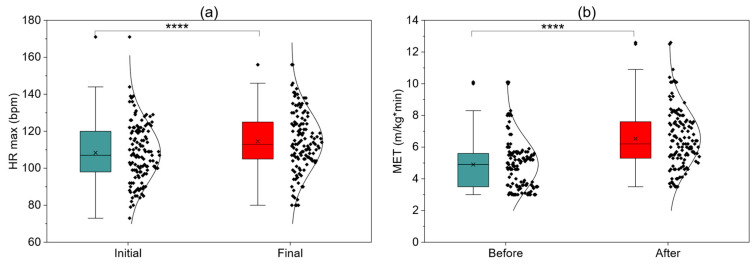

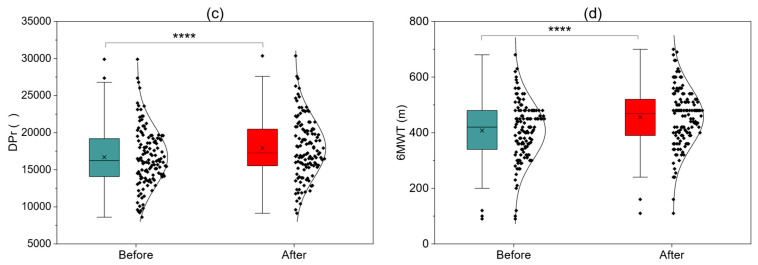
Baseline and final assessments of patients—interval training (**a**) HR max; (**b**) MET; (**c**) DPr; (**d**) 6MWT; ****—*p* < 0.0001.

Figure 3 presents changes in key exercise and hemodynamic parameters in the interval training group before and after completion of 24-session cardiac rehabilitation. In patients performing this protocol, a significant increase in maximum heart rate (HR max) (Figure 3a) and MET value (Figure 3b) was observed, reflecting improved exercise tolerance and metabolic capacity. Double product (DPr) (Figure 3c), an indirect indicator of myocardial oxygen demand, also increased significantly, indicating safe and favorable hemodynamic adaptation to exercise. In the 6MWT (Figure 3d), a clear increase in distance exceeding the minimal clinically important difference was achieved, confirming improved functional capacity. Collectively, the presented parameters confirm that interval training used in patients with lower baseline capacity leads to significant and multidimensional improvement in physical capacity and exercise parameters.

**Figure 4:**
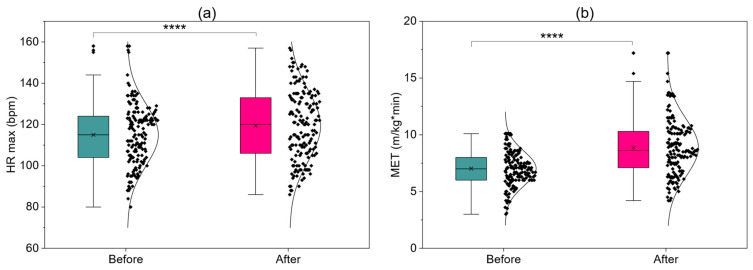

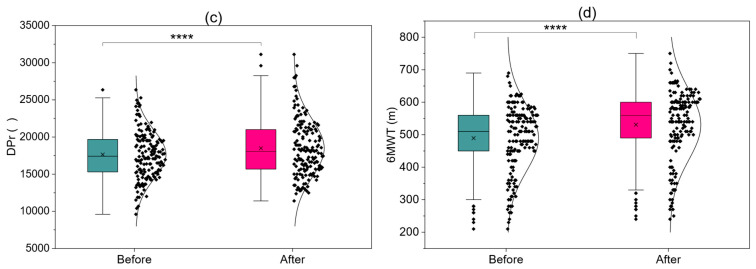
Baseline and final assessments of patients—continuous training (**a**) HR max; (**b**) MET; (**c**) DPr; (**d**) 6MWT; ****—*p* < 0.0001.

Figure 4 presents changes in hemodynamic, metabolic, and functional parameters in the group of patients performing continuous training, assessed before and after a 24-session early cardiac rehabilitation program. A significant increase in maximum heart rate (HR max) (Figure 4a) was observed, reflecting improved exercise tolerance and ability to achieve higher workloads. MET value (Figure 4b) increased very significantly, reaching the highest final values among the analyzed protocols, which results from better baseline exercise tolerance in patients of this group. Double product (DPr) (Figure 4c) also increased after rehabilitation, indicating greater capacity of the cardiac muscle to work at higher loads. Combined with the improvement in heart rate recovery (HRR), observed only in this group, these results indicate a favorable effect of continuous training on autonomic regulation and exercise reserve. In the 6MWT (Figure 4d), a significant increase in distance was achieved, exceeding the minimal clinically important difference, confirming functional improvement in daily activity. Collectively, the presented results indicate that continuous training leads to multidimensional, clinically significant improvement in physical and hemodynamic capacity in patients with higher baseline exercise tolerance.

Our results demonstrate that both training protocols achieve clinically meaningful improvements when appropriately matched to patient baseline capacity and clinical risk. This reflects successful implementation of individualized, guideline-based cardiac rehabilitation within the CSC-Infarct framework.

Patients assigned to interval training (typically older, baseline MET < 6, moderate-high clinical risk) achieved substantial improvements despite lower starting points: MET increased by the highest relative percentage, functional capacity (6MWT) improved by 48.0m (+11.8%), and all cardiovascular parameters showed significant favorable changes. These gains are particularly notable given this group’s baseline characteristics (mean age 65.8 years, higher comorbidity burden including 86.6% with hypertension) and represent clinically significant enhancement in daily functional capacity and quality of life.

Patients assigned to continuous training (typically younger, baseline MET ≥ 6, lower clinical risk) achieved higher absolute final values while maintaining excellent safety profiles: final MET 8.9, VO_2_max 31.0 mL/kg/min, and 6MWT distance 530.9 m. Importantly, only this group demonstrated significant heart rate recovery (HRR) improvement (*p* = 0.0258), suggesting that sustained moderate-intensity exercise may promote more robust parasympathetic reinnervation and autonomic balance restoration in patients with adequate baseline capacity to tolerate this training intensity [50,51,52,53,54].

The key insight from our real-world observational data is not that one training method is inherently superior, but rather that individualized protocol selection based on baseline exercise capacity and clinical risk enables safe, effective rehabilitation across the full spectrum of post-STEMI patients.

Both protocols successfully delivered rehabilitation during the optimal biological timing window (mean 16.8 days, peak remodeling period), with zero serious adverse events across 288 patients spanning ages 35–87 years and baseline MET values 3.0–10.1. This demonstrates that the CSC-Infarct program’s individualized approach to early rehabilitation is both pragmatic and effective in real-world clinical practice.

## 4. Discussion

This study provides evidence for the safety and effectiveness of very early cardiac rehabilitation initiated on average 16.8 ± 3.4 days after STEMI within the Coordinated Specialist Care Program in 288 patients.

The timing of rehabilitation initiation is particularly important from the perspective of remodeling biology. As detailed in the Introduction, the timing of rehabilitation initiation is particularly important from the perspective of remodeling biology. Our mean rehabilitation start time of 16.8 ± 3.4 days represents intervention during the transitional phase between inflammatory and proliferative remodeling, when matrix metalloproteinase activity is elevated [5,6,9,10,11,12,13,14,15]. This timing window may be critical for favorably modulating the remodeling process.

Our results showing no adverse events and good training tolerance in 288 patients support the safety of rehabilitation intervention during this critical period, consistent with previous experimental and clinical evidence [16,17,20,21,25,27].

The CSC-Infarct program, described in detail in the Introduction, ensures earlier rehabilitation access compared to many international systems where rehabilitation typically begins 2–6 weeks post-event [55,56]. Our results confirm that this early care model with mean start time of 16.8 days is associated with good tolerance and significant functional improvement in both training protocols. This aligns with previous Polish studies demonstrating safety and effectiveness of early rehabilitation within CSC-Infarct [8,48,49,57], and broader evidence linking cardiac rehabilitation participation to improved outcomes including 40% reduction in hospitalizations [7].

Our study reflects the real clinical practice of the CSC-Infarct program, where the choice of training protocol is individualized based on the patient’s baseline exercise tolerance [22,23,43,44]. Patients with lower exercise tolerance (MET < 6) receive interval training, while patients with higher tolerance (MET ≥ 6) receive continuous training.

An important finding is that both training methods proved safe and effective for their target populations. Despite significant differences in baseline exercise tolerance (MET 4.9 Vs. 7.0; *p* < 0.001), both groups achieved clinically significant improvement: VO_2_max (interval group: +33.3%, continuous group: +26.2%); distance in 6MWT exceeding the minimal clinically important difference (interval group: +49.0 m [+11.8%], continuous group: +41.5 m [+8.5%]); increase in metabolic capacity (both groups *p* < 0.001); reduction in subjective perception of exertion (both groups *p* < 0.001).

Final values of VO_2_max and MET were higher in the continuous group (31.0 Vs. 22.9 mL/kg/min; 8.9 Vs. 6.5 MET); however, these results should be interpreted in the context of baseline differences. The observed final differences primarily reflect differences in patients’ baseline fitness rather than superiority of one method over another.

It should be noted that there is a difference in the definition of “interval training” between international studies and Polish practice. In international meta-analyses [38,39,40], HIIT refers to very high-intensity training (85–95% HR max) used in stable patients. In the Polish system, “interval” training is dedicated to patients with lower exercise tolerance and includes alternating periods of lower and moderate intensity [40,41].

An important finding is the significant HRR improvement observed only in the continuous training group (from 90.3 to 86.7 bpm, *p* = 0.026). As HRR is a recognized marker of cardiovascular autonomic balance and slower recovery is associated with increased mortality risk [50,51,52], this finding suggests favorable autonomic reinnervation with continuous training. However, HRR is influenced by multiple factors including baseline exercise capacity, age, and pharmacological therapy (particularly β-blockers), therefore our observational design does not permit separation of these effects. The association between continuous moderate-intensity training and improved autonomic balance restoration has been reported previously [52,53,54] and may represent one mechanism through which exercise favorably influences post-infarction remodeling [54,58,59,60].

The relationship between autonomic balance and left ventricular remodeling is intensively studied. Sympathetic overactivity after infarction contributes to adverse remodeling [58,60]. Modulation of autonomic balance through training may be one of the mechanisms of favorable remodeling [54,59]; however, this requires confirmation in studies with direct measurement of cardiac structural parameters.

The 6MWT is a widely used tool for assessing functional capacity, with a minimal clinically important difference of 30–50 m [61,62,63]. This functional assessment, as part of comprehensive multidisciplinary cardiac rehabilitation approach [64], provides valuable information about patients’ real-world exercise capacity. Both groups achieved clinically significant improvement: interval group +48.0 m (11.8%), continuous group +41.5 m (8.5%). These results are consistent with previous observations from our team [8,42,55].

Double product (DPr) is a clinical indicator of myocardial oxygen demand [65]. We observed a significant increase in DPr in both groups (interval: +12.4%, continuous: +9.1%). The simultaneous increase in DPr and exercise tolerance without ischemic symptoms indicates improved cardiac work efficiency and safety of the applied protocols.

We observed a significant reduction in perception of exertion in both groups (interval: from 14.4 to 13.5, continuous: from 13.9 to 12.8; *p* < 0.001 for both). The reduction in score at the same or higher exercise intensity indicates cardiovascular adaptation and improvement in metabolic efficiency [47].

### Limitations

The primary limitation of this study is the significant age difference between training groups. Patients in the interval training group were on average 7.8 years older than those receiving continuous training (65.8 vs. 58.1 years; *p* = 0.0001), which represents a major confounding variable. This age disparity, combined with lower baseline exercise capacity in the interval group (MET 4.9 Vs. 7.0; *p* < 0.0001), resulted from non-randomized, indication-based allocation following standard clinical practice. Since age independently affects exercise capacity, autonomic function, and post-infarction recovery, the observed differences in final outcomes cannot be attributed solely to training modality but reflect both training effects and baseline patient characteristics. Therefore, this study should be interpreted as demonstrating the safety and effectiveness of individualized early rehabilitation rather than comparing the superiority of specific training methods.

Additional limitations include lack of direct remodeling assessment through serial echocardiographic or CMR studies and biomarkers (MMP-2, MMP-9, TIMP-1, NT-proBNP). Future studies should include these parameters along with longer follow-up (6–12 months) to assess sustained benefits and clinical event rates. The single-center design may limit generalizability. The absence of a non-rehabilitation control group, while ethically justified given strong evidence for cardiac rehabilitation benefits (Class I, Level A recommendation) [22,23], prevents direct quantification of rehabilitation effects versus natural recovery. Additionally, detailed data on β-blocker dosage and titration were not systematically collected, limiting ability to control for pharmacological influences on heart rate parameters.

Despite these limitations, this study provides valuable real-world evidence on the safety and effectiveness of very early cardiac rehabilitation (mean 16.8 days post-STEMI) in a large consecutive cohort (N = 288) of post-STEMI patients treated in routine clinical practice. The pragmatic design enhances external validity and applicability to real-world settings.

## 5. Conclusions

Early cardiac rehabilitation initiated on average 16.8 days after STEMI within the CSC-Infarct program demonstrates safety and effectiveness. Both interval and continuous training protocols are associated with significant improvements in cardiorespiratory fitness (VO_2_max, MET), functional capacity (6MWT), and hemodynamic parameters. Observed differences in post-rehabilitation values between training groups should be interpreted in the context of unequal baseline exercise capacity rather than as evidence of superior efficacy of one training modality.

Higher final values of capacity parameters in the continuous training group reflect higher baseline values of this group, which constitute the criterion for assignment to a given protocol, and are not the result of randomized comparison of training modalities.

The improvement in heart rate recovery observed only in the continuous training group suggests potentially different associations of various training protocols with markers of autonomic balance. This requires confirmation in studies with randomization and control of confounding variables.

The CSC-Infarct program ensures early access to rehabilitation during the period of intense remodeling. The impact on the cardiac remodeling process requires confirmation in studies with direct measurement of remodeling parameters (echocardiography, CMR, biomarkers).

Individualization of training modality selection based on baseline exercise tolerance, used in the CSC-Infarct program, may be a safe and pragmatic approach in clinical practice. Therefore, the present results should be interpreted as evidence of the effectiveness and safety of individualized early rehabilitation within the CSC-Infarct program, rather than as a comparative efficacy analysis of training modalities.

## Figures and Tables

**Table 1:** Demographic and clinical characteristics of the study group in the context of early CSC-Infarct rehabilitation.

Parameter	All Group (N = 288)	Interval Training (n = 127)	Continuous Training (n = 161)	*p*-Value
Age (years)	61.5 ± 10.7	65.8 ± 9.2	58.1 ± 10.6	**<0.001** ^1^
Sex (F/M), n (%)	Female: 135 (46.9%)	Female: 66 (52.0%)	Female: 69 (42.9%)	0.156 ^2^
	Male: 153 (53.1%)	Male: 61 (48.0%)	Male: 92 (57.1%)	
BMI (kg/m^2^)	27.9 ± 4.6	28.3 ± 4.7	27.6 ± 4.6	0.260 ^1^
BMI-classification				0.550 ^3^
underweight	2 (0.7%)	0 (0.00%)	2 (1.2%)	
normal weight	98 (34.0%)	41 (32.3%)	57 (35.4%)	
overweight	92 (31.9%)	41 (32.3%)	51 (31.7%)	
class I obesity	71 (24.7%)	31 (24.4%)	40 (24.8%)	
class II obesity	23 (8.0)	13 (10.2%)	10 (6.2%)	
class III obesity	2 (0.7%)	1 (0.8%)	1 (0.6%)	
WHR	1.0 ± 0.2	1.0 ± 0.2	1.0 ± 0.2	0.789 ^1^
LVEF (%)	52.3 ± 9.4	51.0 ± 10.2	53.3 ± 8.6	0.060 ^1^
Time from infarction (days)	16.8 ± 3.4	16.9 ± 3.6	16.7 ± 3.3	0.763 ^1^

Data are presented as Mean ± SD. Abbreviations: BMI—body mass index; WHR—waist-to-hip ratio; LVEF—left ventricular ejection fraction; Full BMI classification according to the World Health Organization (WHO): <18.5—underweight; 18.5 to 24.9—normal weight; 25.0 to 29.9—overweight; 30.0 to 34.9—class I obesity; 35.0 to 39.9—class II obesity; ≥40.0—class III obesity. Left ventricular systolic function LVEF (%) risk classification: ≥50%—low; 36–49%—moderate; ≤35%—high. Type of statistical test used: ^1^—Mann–Whitney U test; ^2^—Chi-square test with Yates’ correction; ^3^—Chi-square test.

**Table 2:** Prevalence of cardiovascular disease risk factors. Presented as number and % of given group.

Parameter	All Group (N = 288)	Interval Training (n = 127)	Continuous Training (n = 161)	*p*-Value
Overweight/obesity	Yes—185 (64.2%)	Yes—85 (66.9%)	Yes—100 (62.1%)	0.470 ^1^
	No—103 (35.8%)	No—42 (33.1%)	No—61 (37.9%)	
Hypertriglyceridemia	Yes—188 (65.8%)	Yes—79 (62.2%)	Yes—109 (67.7%)	0.396 ^1^
	No—100 (34.7%)	No—48 (37.8%)	No—52 (32.3%)	
Smoking	Yes—110 (39.2%)	Yes—44 (34.7%)	Yes—66 (41.0%)	0.328 ^1^
	No—178 (61.8%)	No—83 (65.4%)	No—95 (59.0%)	
Hypertension	Yes—219 (76.0%)	Yes—110 (86.6%)	Yes—109 (67.7%)	**<0.001** ^1^
	No—69 (24.0%)	No—17 (13.4%)	No—52 (32.3%)	
**Diabetes**	Yes—109 (37.9%)	Yes—48 (37.8%)	Yes—61 (37.9%)	0.915 ^1^
	No—179 (62.2%)	No—79 (62.2%)	No—100 (62.1%)	

Type of statistical test used: ^1^—Pearson’s Chi-squared test with Yates’ continuity correction.

**Table 3:** Comparison of hemodynamic, metabolic, and functional parameters before and after early cardiac rehabilitation within CSC-Infarct between interval training and continuous training groups.

Baseline Data				
Parameter	All Group (N = 288)	Interval Training (n = 127)	Continuous Training (n = 161)	*p*-Value Interval vs. Continuous
HR rest	73.5 ± 12.0	73.9 ± 11.6	73.2 ± 12.3	0.516 ^1^
HR max	112.1 ± 15.8	108.4 ± 16.2	115.0 ± 14.9	**<0.001** ^1^
HRR	89.0 ± 13.4	87.4 ± 13.0	90.3 ± 13.7	0.134 ^1^
BP rest systolic	121.8 ± 15.3	123.4 ± 16.9	120.6 ± 13.7	0.155 ^1^
BP rest diastolic	77.3 ± 7.5	76.9 ± 6.8	77.6 ± 8.0	0.271 ^1^
BP max systolic	154.1 ± 19.0	153.9 ± 21.2	154.1 ± 17.1	0.810 ^1^
BP max diastolic	80.6 ± 7.0	80.9 ± 6.4	80.4 ± 7.5	0.871 ^1^
MET	6.1 ± 1.9	4.9 ± 1.6	7.0 ± 1.5	**<0.001** ^1^
Predicted exercise capacity for age (MET)	7.8 ± 1.9	7.1 ± 1.8	8.4 ± 1.8	**<0.001** ^1^
% of age-appropriate exercise capacity (MET)	80.0 ± 24.7	70.8 ± 21.8	87.2 ± 24.5	**<0.001** ^1^
VO_2_max	21.3 ± 6.6	17.2 ± 5.6	24.6 ± 5.4	**<0.001** ^1^
Saturation	97.3 ± 0.7	97.3 ± 0.8	97.4 ± 0.7	0.133 ^1^
DPr	17,222 ± 3601	16,695 ± 3944	17,638 ± 3259	**0.027** ^1^
6MWT	453.4 ± 110.3	407.7 ± 103.0	489.4 ± 102.5	**<0.001** ^1^
Borg rating of perceived exertion	14.1 ± 1.8	14.4 ± 1.5	13.9 ± 1.9	**0.008** ^1^
Final data				
HR rest	73.2 ± 11.1	72.0 ± 9.5	74.2 ± 12.2	0.200 ^1^
HR max	117.3 ± 16.9	114.5 ± 16.4	119.4 ± 17.0	**0.025** ^1^
HRR	87.2 ± 17.1	87.9 ± 15.1	86.7 ± 18.5	0.538 ^2^
BP rest systolic	121.6 ± 15.2	123.6 ± 16.0	120.0 ± 14.4	0.089 ^1^
BP rest diastolic	76.6 ± 7.3	77.2 ± 7.0	76.2 ± 7.4	0.470 ^1^
BP max systolic	155.4 ± 18.6	155.7 ± 19.7	155.3 ± 17.8	0.943 ^1^
BP max diastolic	78.6 ± 7.1	79.1 ± 7.4	78.3 ± 6.8	0.613 ^1^
MET	7.8 ± 2.5	6.5 ± 1.9	8.9 ± 2.5	**<0.001** ^1^
% of age-appropriate exercise capacity (MET)	102.0 ± 27.4	94.6 ± 25.3	107.8 ± 27.7	**<0.001** ^1^
VO_2_max	27.4 ± 8.8	22.9 ± 6.5	31.0 ± 8.8	**<0.001** ^1^
Saturation	97.7 ± 0.9	97.5 ± 0.9	97.8 ± 1.0	0.133 ^1^
DPr	18,233 ± 3953	17,916 ± 4036	18,483 ± 3880	0.337 ^1^
6MWT	497.7 ± 113.1	455.6 ± 104.3	530.9 ± 108.9	**<0.001** ^1^
Borg rating of perceived exertion	13.1 ± 0.9	13.5 ± 0.9	12.8 ± 0.9	**<0.001** ^1^

Data are presented as Mean ± SD. Abbreviations: HR—heart rate; HRR—heart rate recovery; BP—blood pressure; MET—metabolic equivalent; VO_2_max—maximum oxygen uptake; DPr—double product; 6MWT—6 min walk test. Type of statistical test used: ^1^—Mann–Whitney U test; ^2^—Welch’s *t*-test.

**Table 4:** Baseline vs. final assessments (*p*-value).

Parameter	All Group (N = 288)	Interval Training (n = 127)	Continuous Training (n = 161)
HR rest	0.281 ^1^	**0.039** ^1^	0.719 ^1^
HR max	**<0.001** ^1^	**<0.001** ^2^	**<0.001** ^1^
HRR	0.252 ^1^	0.290 ^1^	**0.026** ^1^
BP rest systolic	0.834 ^1^	0.805 ^1^	0.551 ^1^
BP rest diastolic	0.150 ^1^	0.893 ^1^	**0.047** ^1^
BP max systolic	0.420 ^1^	0.548 ^1^	0.593 ^1^
BP max diastolic	**0.001** ^1^	**0.014** ^1^	**0.002** ^1^
MET	**<0.001** ^1^	**<0.001** ^1^	**<0.001** ^1^
% of age-appropriate exercise capacity (MET)	**<0.001** ^1^	**<0.001** ^1^	**<0.001** ^1^
VO_2_max	**<0.001** ^1^	**<0.001** ^1^	**<0.001** ^1^
Saturation	**<0.001** ^1^	**<0.001** ^1^	**<0.001** ^1^
DPr	**<0.001** ^1^	**<0.001** ^1^	**<0.001** ^1^
6MWT	**<0.001** ^1^	**<0.001** ^1^	**<0.001** ^1^
Borg rating of perceived exertion	**<0.001** ^1^	**<0.001** ^1^	**<0.001** ^1^

Abbreviations: HR—heart rate; HRR—heart rate recovery; BP—blood pressure; MET—metabolic equivalent; VO_2_max—maximum oxygen uptake; DPr—double product; 6MWT—6 min walk test. Type of statistical test used: ^1^—Wilcoxon signed rank test with continuity correction; ^2^—Student’s *t*-test.

## Data Availability

Data supporting the conclusions of this study are available from the corresponding authors upon reasonable request. Data cannot be made publicly available due to patient privacy protection in accordance with GDPR (EU General Data Protection Regulation 2016/679).

## Abbreviations

The following abbreviations are used in this manuscript:

KOS	Poland’s Coordinated Specialist Care Program
HRR	heart rate recovery
MMPs	matrix metalloproteinases
LVEF	left ventricular ejection fraction
NFZ	National Health Fund
ACS	acute coronary syndrome
6MWT	6 min walk test
PCI	percutaneous coronary intervention
DPr	double product
